# Supplementary material for: EWS-FLI1 regulates and cooperates with core regulatory circuitry in Ewing sarcoma
Source: Nucleic Acids Res. 2020 Oct 20;48(20):11434–51. doi: 10.1093/nar/gkaa901 (PMC7672457; doi:10.1093/nar/gkaa901)
Supplement: gkaa901_Supplemental_Files [file gkaa901_supplemental_files.zip › Supplementary Figure S1-S5 and Table S1-S3.pdf]

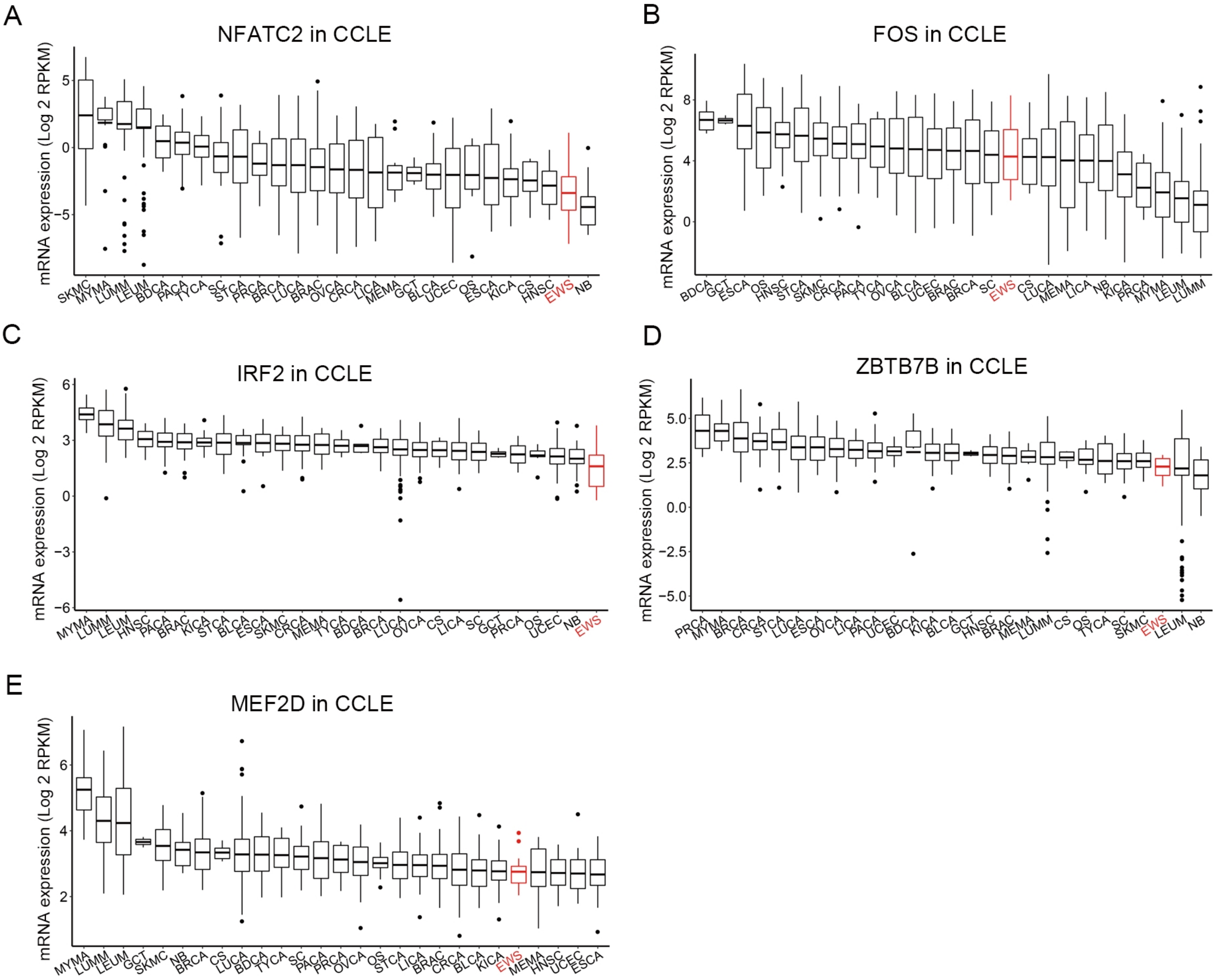

Supplementary Figure S1. The mRNA levels of candidate TFs (NFATC2, FOS, IRF2, ZBTB7B and MEF2D) in CCLE database.

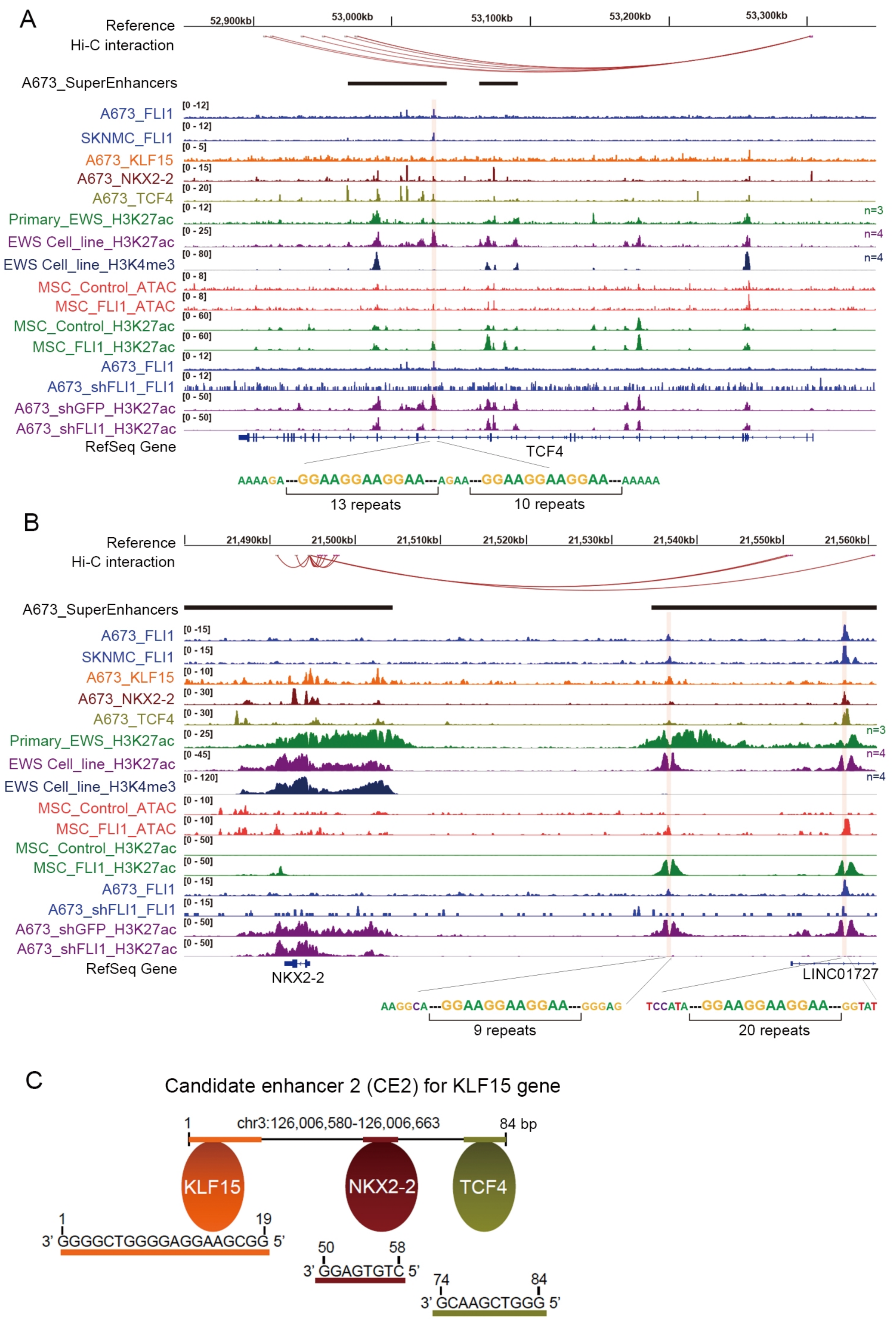

Supplementary Figure S2. Co-binding peaks of EWS-FLI1 and CRC TFs at the genomic loci of TCF4 and NKX2-2. IGV plots of ChIP-Seq showing co-occupancy of EWS-FLI1 and CRC TFs at the superenhancers and promoters of TCF4 (A) and NKX2-2 (B). The tracks were showing the overlapping signals of 3 primary Ewing sarcoma tissues and 4 Ewing sarcoma cell lines. Hi-C interactions were re-analyzed from the data of SKNMC cell line downloaded from ENCODE database; H3K27ac, H3K4me3 and EWS-FLI1 ChIP-Seq data were retrieved from GEO (GSE61953). ATAC-Seq and ChIP-Seq profiles at superenhancers of TCF4 and NKX2-2 in the presence and absence of either EWS-FLI1 overexpression or knockdown. Data were retrieved from GEO (GSE61953). (C) The TF spacing in candidate enhancer 2 (CE2) for KLF15.

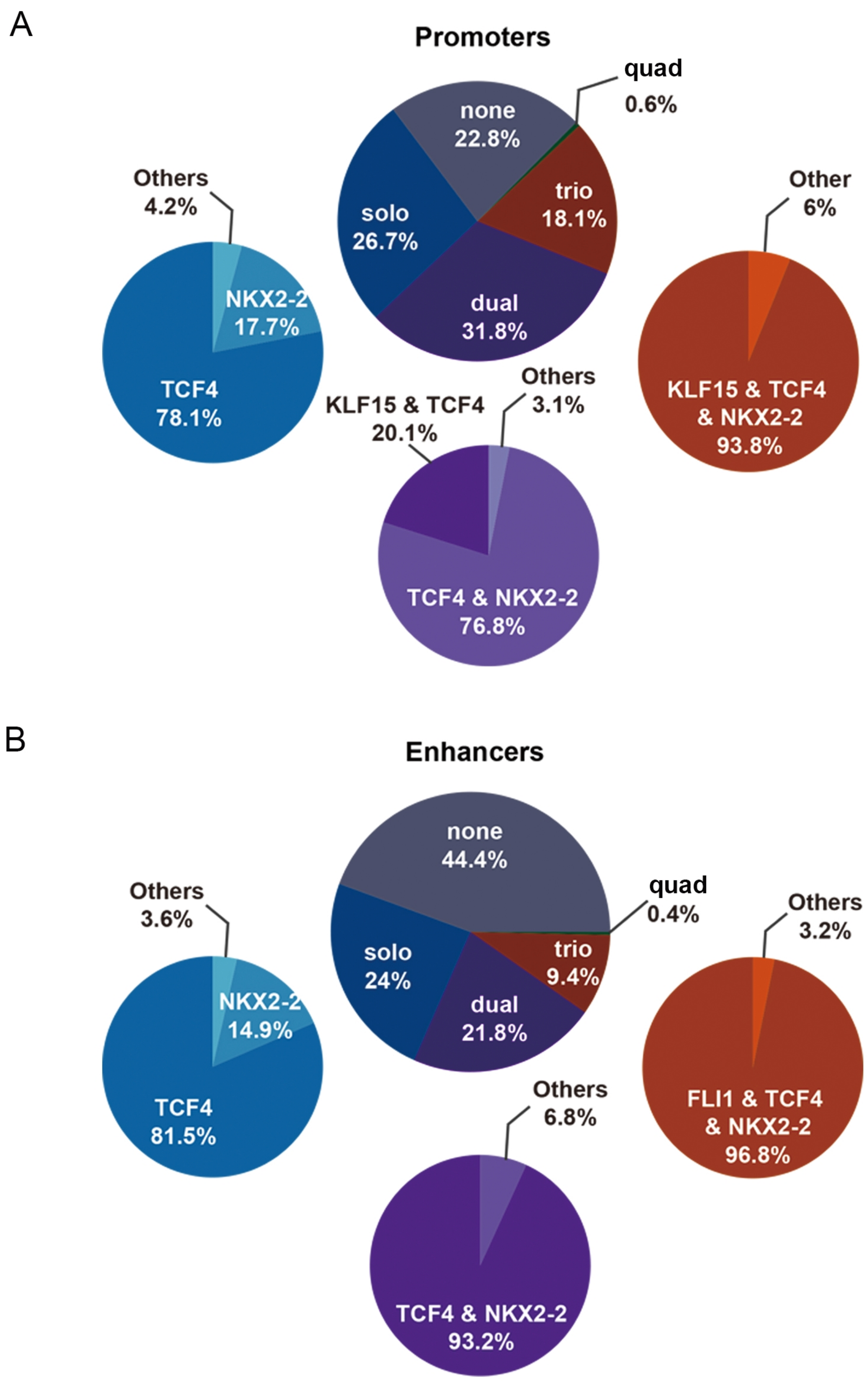

Supplementary Figure S3. Pie charts of the factions of combinatorial binding patterns of EWSFLI1 and three CRC TFs in both enhancers and promoters.

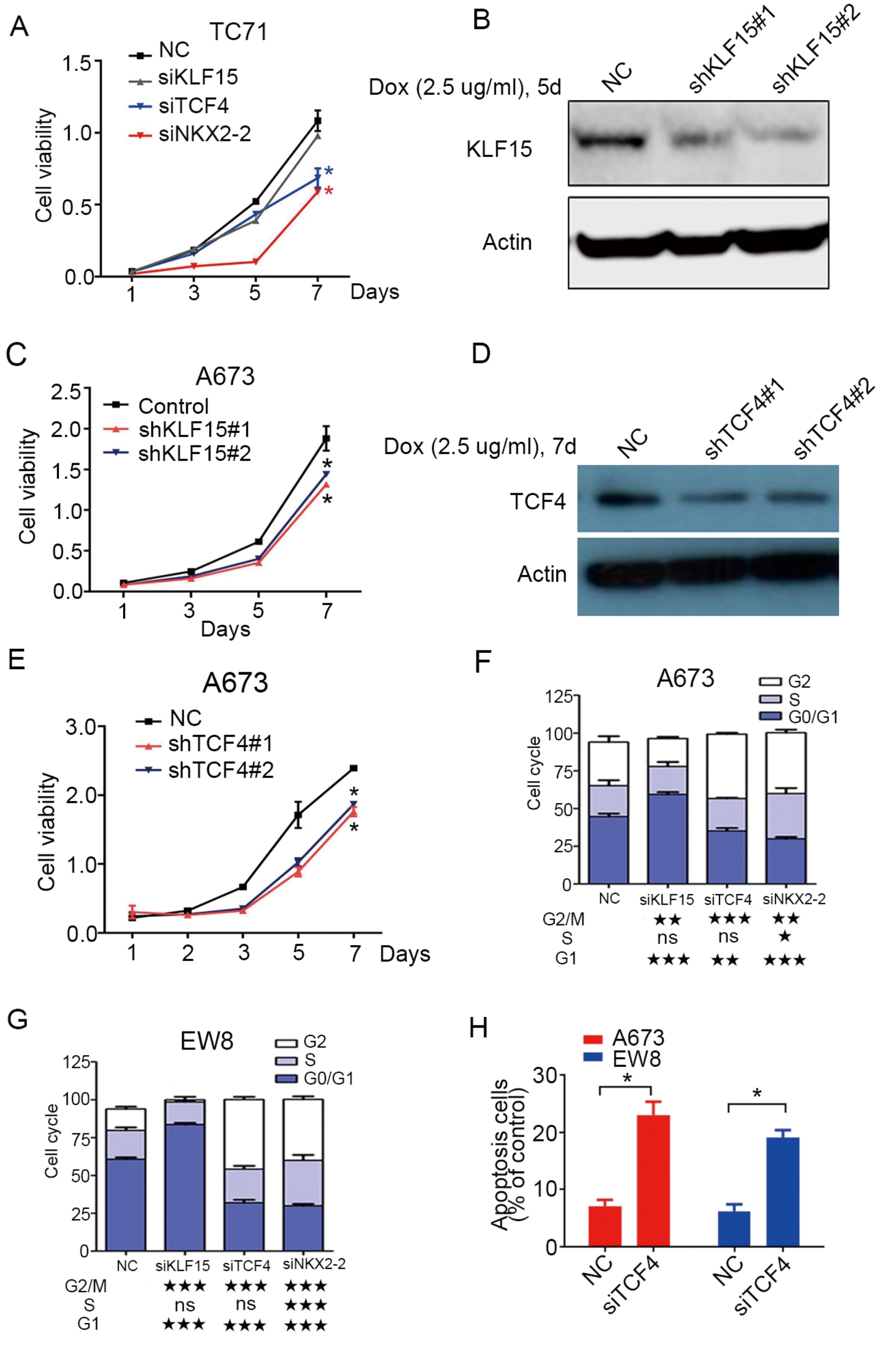

Supplementary Figure S4. The cancer-promoting functions of KLF15 and TCF4 in Ewing sarcoma cells. (A) Silencing of each of three CRC TFs by siRNAs decreased cell proliferation in TC71 cells. (B-E) Knockdown of KLF15 (B, C) or TCF4 (D, E) by inducible shRNAs decreased cell proliferation in A673 cells. (F, G) Knockdown of three CRC TFs by individual siRNAs increased cell-cycle arrest in Ewing sarcoma cell lines. (H) Silencing of TCF4 by siRNAs induced cell apoptosis in A673 and EW8 cells. NC, non-targeting control siRNA or shRNA. Cells were stained with Annexin V/PI. Mean ± s.d. are shown, n = 6. *, $P < 0.05$; **, $P < 0.01$.

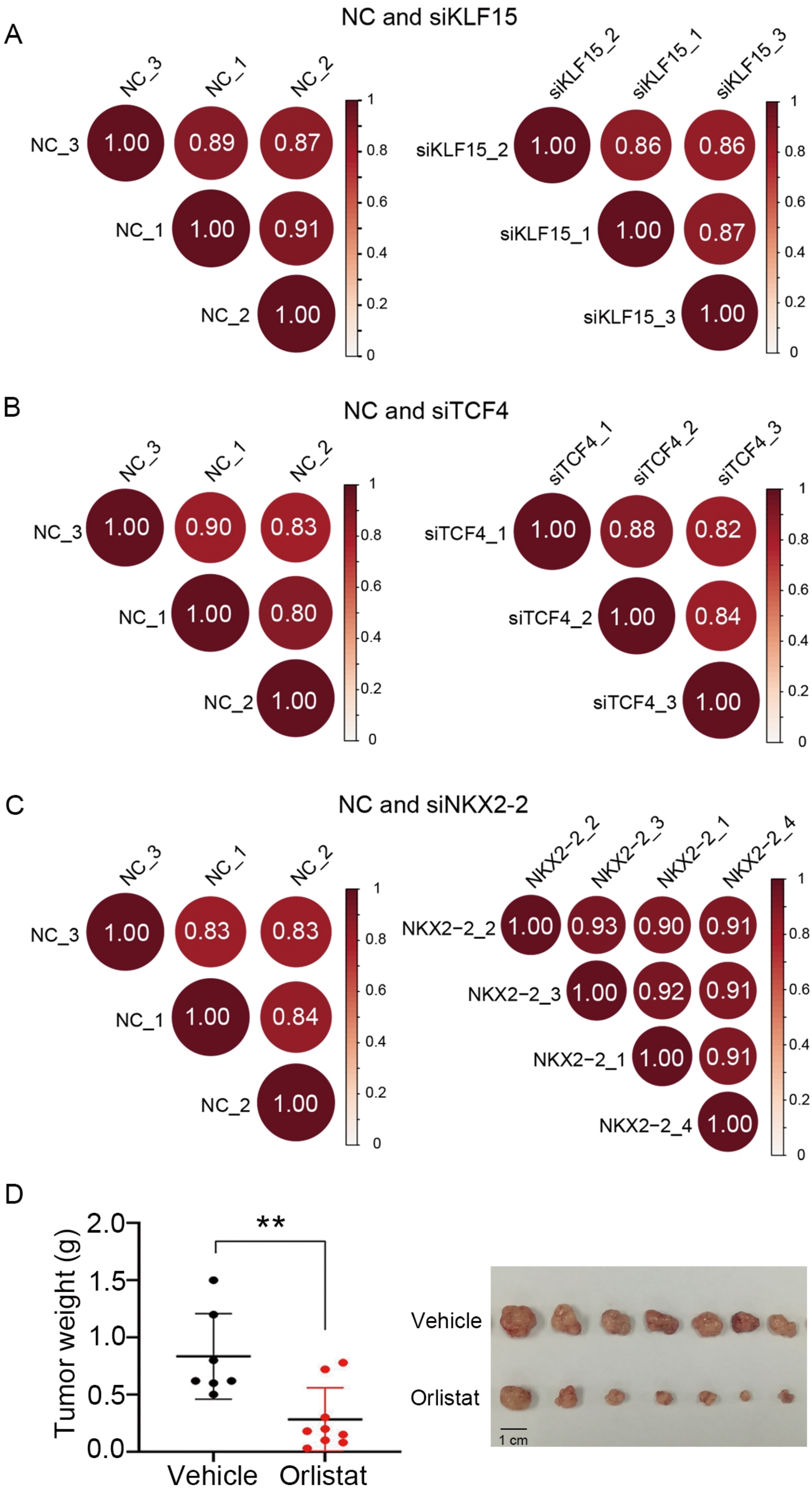

Supplementary Figure S5. (A-C) Pearson correlation plots between replicates of LC-MS/MS-based lipidomic samples from each group. (D) Orlistat treatment suppressed xenograft growth in vivo. Tumor weights and images of resected tumors from both groups are shown. Two-way ANOVA Dunnett's multiple comparisons test. n = 6. *, P< 0.5, **, P < 0.01; ***, P < 0.001.

Supplementary Table S1: siRNA sequences

Target	sense（5'-3'）	antisense（5'-3'）
siFLI1 #1	GGGAGUAUGACCACAUGAATT	UUCAUGUGGUCAUACUCCCTT
siFLI1 #2	GCCAUAAAGGAGUACAGCUTT	AGCUGUACUCCUUUAUGGCTT
siKLF15 #1	GCAUCUUGGACUUCCUAUUTT	AAUAGGAAGUCCAAGAUGCTT
siKLF15 #2	GCUUGCCCGAGUUUCCUUUTT	AAAGGAAACUCGGGCAAGCTT
siTCF4 #1	GGGACAUGCAUGGAAUCAUTT	AUGAUUCCAUGCAUGUCCCTT
siTCF4 #2	CUCAUCGUCUCCUAAUUAUTT	AUAAUUAGGAGACGAUGAGTT
siNKX2-2 #1	CCUGCCGGACACCAACGAUTT	AUCGUUGGUGUCCGGCAGGTT
siNKX2-2 #2	GCACCGAGGGCCUUCAGUATT	UACUGAAGGCCCUCGGUGCTT
siSCD #1	GACGAUAUCUCUAGCUCCUTT	AGGAGCUAGAGAUAUCGUCTT
siSCD #2	GGUUGAAUAUGUCUGGAGATT	UCUCCAGACAUAUUCAACCTT
siFASN #1	GGACCUGUCUAGGUUUGAUTT	AUCAAACCUAGACAGGUCCTT
siFASN #2	CCCAGGCUGAAGUUUACAATT	UUGUAAACUUCAGCCUGGGTT
siRREB1 #1	GAGCGAACCUUCACCUUGATT	UCAAGGUGAAGGUUCGCUCTT
siRREB1 #2	GACCUAUCUUCCAUCAACATT	UGUUGAUGGAAGAUAGGUCTT
siSPTLC1 #1	GAUCUGAUCUUACAGUCAATT	UUGACUGUAAGAUCAGAUCTT
siSPTLC1 #2	GGAUUGUUGGAUAACCCUATT	UAGGGUUAUCCAACAAUCCTT

Supplementary Table S2: The sequences of sgRNA

sgRNA	Sequence (5'-3')
KLF15-sgRNA-CE1-1	CACCGATTGGCCCACCAGCACCCTGGTTT
KLF15-sgRNA-CE1-2	CACCGAGTACGTTCTGCTTTGGAGAGTTT
KLF15-sgRNA-CE2-1	CACCGAGTCCTGATTCCACGATAGGGTTT
KLF15-sgRNA-CE2-2	CACCGAGATAATGGAGTGCGTGAGCGTTT

Supplementary Table S3: SE-associated TFs in EWS

**SE-associated TFs in EWS**							
RERE	FOXK2	ZEB2	ZBTB16	RREB1	SALL2	BARX2	NPAS3
ZNF532	EBF3	ATF7	NFIC	IKZF2	ZBTB7B	TEF	ZNF219
IRF2	FOXP4	FOXK1	NR2F2	SOX1	RCOR1	AEBP2	MEF2D
MEIS1	TOX2	SMAD3	HLF	YY1	CIC	SCXB	ZBTB2
RXRA	ARID5B	GLIS2	KLF15	ATF4	BARHL2	RFX3	POU1F1
NFIA	TCF4	ZFHX3	TCF12	NFATC2	FIZ1	TP63	FEZF1
BCL11B	ALX4	GLI3	FOS	VAX1	ID3	ID1	NFIB
NFIX	ZBTB7A	PAX7	ARX	NKX2-2	TEAD2	ZBTB20	
ARID1A	MNT	ZIC5	MSC	PAX3	ETV1	DLX2	
SREBF2	SOX6	ZNF217	ADNP	CBFB	ETV3	DLX6-AS1	
